# T. S. Cobbold, M.D., F.R.S.

**Published:** 1886-04

**Authors:** 


### T. S. COBBOLD, M.D., F.R.S.

We regret to announce, as we go to press, the news of the death of the celebrated helminthologist, T. S. Cobbold, M.D., F.R.S., formerly Professor in the Royal Veterinary College, with whose writings our readers are familiar.

